# Clarification of Temperature Field Evolution in Large-Scale Electric Upsetting Process of Ni80A Superalloy through Finite Element Method

**DOI:** 10.3390/ma15186358

**Published:** 2022-09-13

**Authors:** Jiang Zhao, Guo-Zheng Quan, Yu-Qing Zhang, Jian-Sheng Zhang

**Affiliations:** 1 Chongqing Key Laboratory of Advanced Mold Intelligent Manufacturing, School of Material Science and Engineering, Chongqing University, Chongqing 400044, China; 2 State Key Laboratory of Materials Processing and Die & Mould Technology, Huazhong University of Science and Technology, Wuhan 430074, China; 3 Chongqing Jiepin Technology Co., Ltd., Chongqing 400050, China

**Keywords:** electric upsetting, temperature field, numerical simulation, nickel-based alloy

## Abstract

Electric upsetting has been widely employed to manufacture the preformed workpiece of large-scale exhaust valves. The temperature field in the electric upsetting process plays an important role in microstructure evolution and defect formation. In order to uncover the temperature evolution in a larger-scale electric upsetting process, the electric-thermal-mechanical multi-field coupling finite element model was developed to simulate the electric upsetting forming process of Ni80A superalloy. The temperature distribution characteristics and their formation mechanisms under different stages were analyzed systematically. Results indicate that at the preheating stage, the billet temperature increases from 20 °C to 516.7 °C, and the higher temperature region firstly appears at the contact surface between billet and anvil due to the combined effects of contact resistance and volume resistance. With increasing preheating time, the higher temperature region is transferred to the interior of the billet because the contact resistance is reduced with increasing temperature. As for the forming process, the billet is gradually deformed into an onion shape. The highest billet temperature increases to 1150 °C and keeps relatively constant. The high temperature region always appears at the neck of the onion due to the relatively higher current density at this place. It enlarges continuously in the primary stage and intermediate stage, and then decreases at the stable deformation stage. The low temperature regions lie in the contact surface and the outer surface of the onion because a lot of heat is lost to the anvil and surroundings through thermal conduction and radiation. Finally, the established finite element model was verified by an actual electric upsetting experiment. The average relative error between simulated temperatures and experimental ones was estimated as 7.54%. The longitudinal and radial errors between simulated onion shape and the experimental one were calculated as 1.38% and 2.70%, respectively.

## 1. Introduction

**Figure 1:**
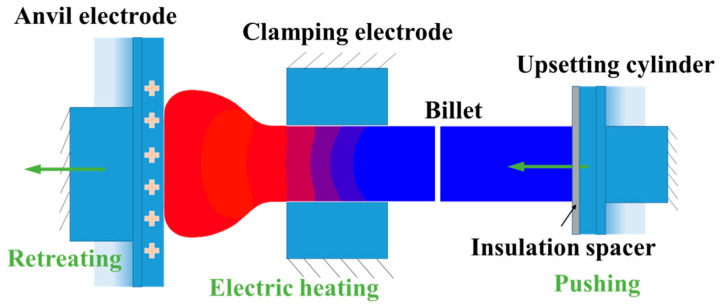
Schematic illustration of electric upsetting process.

Nickel-based alloys have been extensively applied in aviation engines, turbines and marine diesel engines in virtue of their superior strength and strong creep resistance at elevated service temperature [1,2]. As a nickel-based heat-resistant alloy, Ni80A superalloy (UNS N07080) has been widely used to manufacture the exhaust valve of marine diesel engines [3,4,5]. Due to its high content of alloying elements, the thermal deformation behaviors of Ni80A superalloy are sensitive to thermal processing parameters including strain, strain rate and temperature, and its effective deformation parameter range with stable plastic deformation is very narrow [6,7]. Moreover, the exhaust valve is a typical disc-rod component with a significantly large difference in diameter between rod and disc, and the forming of the exhaust valve needs to deform a billet with a high length-to-diameter ratio of up to 20 [8]. It is extremely difficult to directly deform the billet using traditional thermal-processing processes such as die forging. Electric upsetting, as an electrically assisted forming process with the advantages of isostatic pressing and high efficiency, is suitable to deform the long-rod billet for obtaining the preformed workpiece of an exhaust valve. The electric upsetting process can be schematically illustrated as Figure 1.

In a certain electric upsetting process, a long-rod billet is sandwiched between an anvil electrode and an upsetting cylinder, and the stem of the billet is clamped by a pair of clamping electrodes. A low-voltage and high-amperage direct current with step change is loaded on the anvil, and the clamping electrodes are grounded. For the large-scale electric upsetting process, the approximate values of voltage and amperage are 5 V and 20 KA, respectively. In addition, an insulation spacer is placed between the billet and the upsetting cylinder. Thus, the current will flow from the anvil to the billet, and then to the clamping electrodes. The part of the billet with current flowing will be heated by self-resistance and contact resistance. An upsetting force with step change is loaded on the right end surface of the billet by the upsetting cylinder. Under the coupling effects of upsetting force and heating current, that portion of the billet is heated locally and begins to enlarge. Meanwhile, the anvil electrode retreats with a specific speed to make space for the deformed billet. As this process continues, a large portion of the billet material will be formed into a desirable shape.

**Figure 2:**
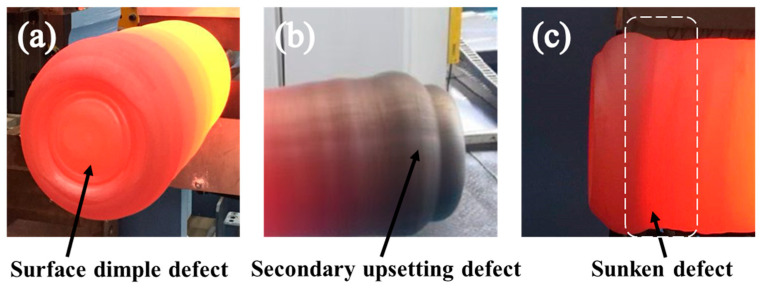
The typical geometric defects formed in the electric upsetting process: (**a**) Surface dimple defect (**b**) Secondary upsetting defect and (**c**) Sunken defect.

It is well known that the properties of an exhaust valve mainly depend on its microstructures, which to a great extent are determined by the grain size in the electric upsetting process [9,10]. The grain size in thermal deformation of Ni80A superalloy is mainly governed by the competition between the grain refinement mechanism of dynamic recrystallization and the grain coarsening mechanism of grain growth. For the large-scale electric upsetting process with long duration time and high deformation temperature, the effect of grain refinement generally cannot counteract the function of grain growth [8]. The final grain size and its homogeneity are greatly influenced by the thermal history of the material [11]. Sun et al. [12] indicate that the forming temperature needs to be strictly controlled in a suitable range, so as to avoid overheating and burning the microstructures. Apart from the role in microstructure evolution, the temperature field in the electric upsetting process has more significant impact on the plastic deformation of a billet. It has been proven that the formation of many typical geometric defects in electric upsetting, such as the secondary upsetting defect [13], surface dimple defect [14], and sunken defect [8], may be induced by abnormal temperature distribution. These geometric defects are indicated in Figure 2. Furthermore, due to the lack of understanding of the formation mechanisms of temperature distribution in large-scale electric upsetting, it is generally impossible to design the electric-upsetting parameters. Up till now, there have been few systematic studies on the temperature evolution in the electric upsetting process, especially for the inner mechanisms causing such temperature distribution. Therefore, it is a significant issue to clarify the distribution and evolution of the temperature field in a large-scale electric upsetting process, so as to guide the actual electric upsetting production for obtaining the desired preformed workpiece of a large-scale exhaust valve.

Nevertheless, the electric upsetting process is essentially a complicated electric-thermal-mechanical multi-field coupling issue, in which various thermal-related mechanisms involving electric heating, heat transfer and thermal radiation coexist. It is extremely difficult to directly observe and predict the temperature field evolution in a practical electric upsetting process. The finite element simulation method (FEM) provides a solution. It can not only consider the coupling effects of an electric-thermal-mechanical multi-field, but also reveal the dynamic evolution process of the temperature field in temporal and spacial domains [15,16]. Biba et al. [17] constructed a thermal-mechanical coupling finite element model to simulate the temperature field and material flow of billet in the electric upsetting process. However, the electric upsetting parameters applied in his model are constant. For the large-scale electrical upsetting, the multi-stage variable loading parameters are generally adopted. Nuasri et al. [18], Jeong et al. [11] and Quan et al. [13] simulated the electric upsetting processes of AISI 1045 steel, Ni80A alloy and 3Cr20Ni10W2 high-alloy using variable loading parameters, respectively. Furthermore, Zhang et al. [8] developed the FEM model of the large-scale electric upsetting process to study the deformation and grain size evolution of a billet, and concluded that billet grain size is very sensitive in the high temperature region. Nevertheless, there is lack of systematic analysis of the evolution of the high temperature region. The above works have been dedicated to obtaining the desired electric upsetting workpiece by changing the processing parameters for achieving the adjustment of temperature distribution and plastic deformation. However, it is usually difficult to design reasonable parameters to achieve the desired temperature distribution. Therefore, we studied the formation mechanisms of the electric upsetting temperature field, intending to lay the foundation for future work on finding new possible ideas for adjusting the temperature distribution of electric upsetting.

In this work, a FEM model of a large-scale electric upsetting process was developed, in which a series of thermal-related behaviors involving electric heating, thermal conduction and thermal radiation were taken into account. The distribution and evolution of the temperature field at different stages of the large-scale electric upsetting process of Ni80A superalloy were uncovered. The location and size of the high temperature region were analyzed, and the formation mechanisms of such temperature distribution characteristics were discussed in detail. Finally, the established FEM model was verified by an actual electric upsetting experiment.

## 2. Finite Element Model of Electric Upsetting Process

### 2.1. Electro-Thermal-Mechanical Coupling Analysis Method

**Figure 3:**
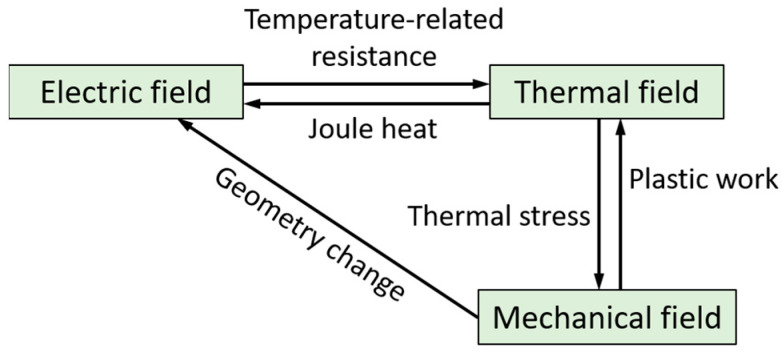
Schematic illustration of the complicated interrelationships between electric, thermal and mechanical fields.

Electric upsetting can be regarded as a current-heating forming process, involving electric heating, heat conduction and thermal-plastic deformation. The finite element simulation of an electric upsetting process can be treated as an electric-thermal-mechanical multi-field coupling problem. It can be realized on many commercial finite-element simulation platforms, such as Deform, Marc, Qform, etc. In order to develop the electric-upsetting FEM model, it is necessary to grasp the complex interactions between electric, thermal and mechanical fields, and then establish quantitative equations to describe them. Here, the coupling relationships among the three fields are schematically described in Figure 3. It is noted from Figure 3 that the coupling relationship between electric field and mechanical field is a one-way street, i.e., the geometric variation in the billet unidirectionally affects the distribution of the electric field. By contrast, the interactions between electric and thermal fields, and between thermal and mechanical fields, are bidirectional. Thus, the electric-thermal-mechanical coupling problem can be treated as a combination of electric-thermal analysis and thermal-mechanical coupling analysis. For the electric-thermal coupling analysis, Ohm’s law is used to calculate the electric parameters, and Joule’s law can be applied for describing the electric heating process. As for the thermal-mechanical coupling module, heat conduction among objects and environment, heat generation by plastic deformation and friction, as well as the influence of temperature on flow stress of material need to be taken into account. The main control equations of electric-thermal-mechanical coupling analysis can be summed up as Equation (1) [19],

(1) $$\{\begin{array}{l} K^{E}(T)V=I\\ Q^{E}=VI\\ C^{T}(T)\dot{T}+K^{T}(T)T=Q^{E}+Q^{P}+Q^{F}-Q^{L}\\ M\ddot{u}+D\dot{u}+K^{M}(T,u,t)u=F+F^{T} \end{array}$$

where *V* represents voltage, *I* is current and *K^E^*(*T*) represents the electric conductivity dependent on temperature; *Q^E^* represents Joule heat. *K^T^*(*T*) and *C^T^*(*T*) represent the temperature-dependent thermal conductivity and specific heat, respectively; *T*, *u* and *t* represent temperature vector, displacement vector and time, respectively; *Q^P^* represents the heat from plastic work; *Q^F^* represents the heat generated by friction; *Q^L^* represents the heat loss;. *M* is mass matrix; *D* represents damping coefficient; *K^M^*(*T*,*u*,*t*) represents stiffness matrix; and *F* and *F^T^* represent external force and thermal stress, respectively.

It must be pointed out that the resistance heat consists of two parts. One is volume-resistance heating, and the other comes from contact-resistance between billet and anvil. Both of them follow Joule’s law. The Joule heat generated by contact resistance is conducted to the billet and anvil proportionally to their heat conductivity. Then, the final expression of Joule heat *Q^E^* is shown as Equation (2) [13],

(2) $$\{\begin{array}{l} Q^{E}=Q_{V}^{E}+Q_{C}^{E}\\ Q_{V}^{E}=I_{n}^{2}R_{V}\\ Q_{C}^{E}=\frac{k_{b}}{k_{b}+k_{a}}I_{n}^{2}R_{C} \end{array}$$

where $Q_{C}^{E}$ and $Q_{V}^{E}$ represent the Joule heat generated by contact-resistance and volume-resistance, respectively; *I_n_* represents the nodal current going through the contact surface and billet; *R_V_* and *R_C_* represent the volume-resistance of the billet, and the contact-resistance between billet and anvils, respectively; and *k*_*a*_ and *k*_*b*_ represent the heat conductivity values of anvil and billet, respectively.

The heat generation owing to plastic work can be estimated by Equation (3) [13],

(3) $$Q^{p}=\beta \bar{\sigma }\dot{\bar{\varepsilon }}$$

where *β* represents the conversion rate of plastic work to heat, $\bar{\sigma }$ is equivalent stress, and $\dot{\bar{\varepsilon }}$ is equivalent strain rate.

The heat generated by friction can be calculated by Equation (4) [13],

(4) $$Q^{F}=\int u\left|\Delta v\right|dt$$

where *u* represent the friction coefficient, and Δv is the relative sliding velocity.

Furthermore, the heat loss due to heat conduction and thermal radiation can be expressed as Equation (5) [13],

(5) $$\{\begin{array}{c} Q^{L}=Q_{C}^{L}+Q_{S}^{L}+Q_{R}^{L}\\ Q_{C}^{L}=h_{c}A_{c}(T_{b}-T_{a})\\ Q_{S}^{L}=h_{s}A_{s}(T_{b}-T_{s})\\ Q_{R}^{L}=fA_{r}(T_{b}{}^{4}-T_{s}{}^{4}) \end{array}$$

where $Q_{C}^{L}$, $Q_{S}^{L}$ and $Q_{R}^{L}$ represent the heat loss due to heat conduction between anvil and billet, heat transfer between billet and surroundings, and thermal radiation between billet and surroundings, respectively; *h*_c_ and *h*_s_ represent the heat conduction coefficient; *f* is thermal radiation coefficient; *A_c_* is the area of contact surface; *A_s_* is the area exposed to surroundings; *A_s_* is the heat radiation area on the billet; and *T_b_*, *T_a_* and *T_s_* represent the temperatures of billet, anvil and surroundings, respectively.

**Figure 4:**
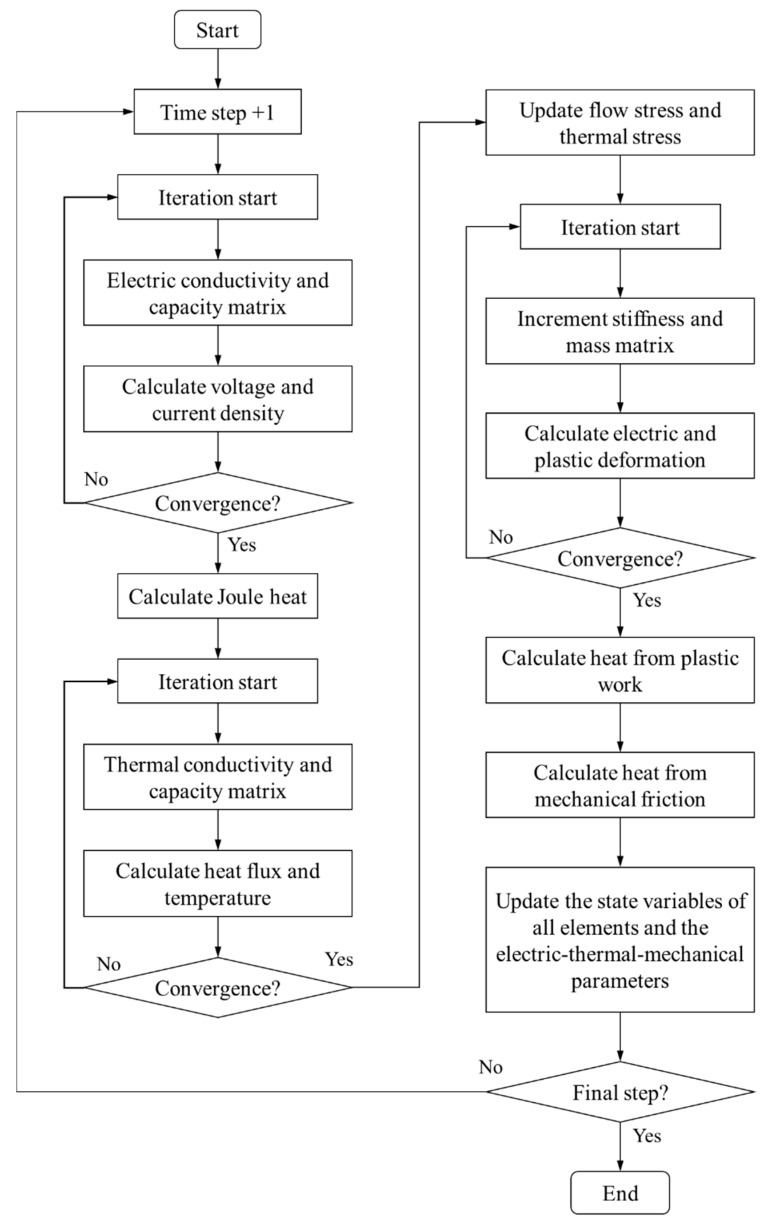
Computing procedures of electric-thermal-mechanical multi-field coupling analysis program.

For a given electric-thermal-mechanical multi-field coupling analysis, the electric field, thermal field and mechanical field will be solved in turn. All of these matrix equations will be iterated many times until convergence. The computing procedures can be schematically described as in Figure 4. It must be noted that the material parameters are updated at the end of each time step.

### 2.2. Development of FEM Model of Electric Upsetting Process

**Figure 5:**
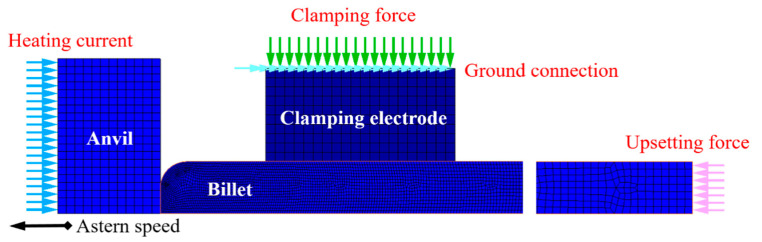
Half-symmetry 2D finite element model of electric upsetting process.

Based on the solving procedures of electric-thermal-mechanical multi-field coupling analysis, the FEM model of electric upsetting can be developed on the solving platform MSC marc. Here, a half-symmetry 2D finite element model was constructed, as shown in Figure 5. A four-node quadrilateral element was adopted to mesh the billet, anvil and clamping electrode. The billet was defined as an elastic-plastic body, while the anvil and clamping electrode were set as rigid bodies. Ni80A superalloy was assigned to the billet, and Titnaium-Zirconium-Molybdenum (TZM) alloy was assigned to the clamping electrode and anvil. The chemical composition and the main properties of the alloys are shown in Table 1, Table 2 and Table 3. The upsetting force was loaded on the right end-surface of the billet. Current density, i.e., current per unit area, was loaded on the left end-surface of the anvil. The potential on the outer surface of the clamping electrode was set as zero, so as to simulate the grounding condition. The contact relations between billet and anvil, and between billet and clamping electrode were considered. For the thermal conductivity and electric resistivity of these objects, as well as their heat transfer coefficient and contact electrical conductivity between them, we can refer to the literature [8]. Macdougall suggests that the conversion efficiency of plastic work to heat is between 0.8 and 1 [20]. Here, it was specified as 0.9. Ambient temperature and the initial temperature of these objects were set as room temperature (20 °C). The friction coefficient was set as 0.3 [8]. Flow stress data of Ni80A superalloy were quoted from reference [21].

**Figure 6:**
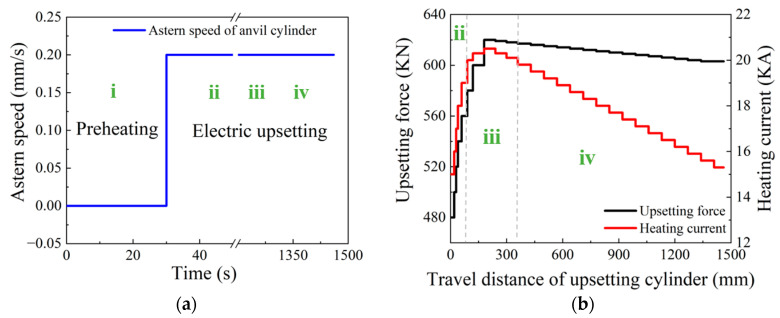
Loading path of electric upsetting processing parameters: (**a**) astern speed of anvil cylinder, and (**b**) upsetting force and heating current. (i, ii, iii and iv represent the four stages of electric upsetting process).

By following the design principles of parameter loading path mentioned in our previous work [8], the upsetting force, heating current and astern anvil speed were set, and the results are shown as Figure 6. It should be noted that the upsetting force and heating current vary with the travel distance of the upsetting cylinder. In order to realize the special loading mode in FEM simulation, the subroutines (“flux.f”, “forcem.f”) were adopted and developed. The operation principle of the two subroutines is as follows: For each time step, the travel distance of the right end-surface of the billet will be extracted first. Then, the heating current and upsetting force corresponding to the present upsetting displacement can be determined and applied in the electric upsetting FEM simulation. The two subroutines will be called at the beginning of each time step.

According to the characteristics of parameter loading path, as in Figure 6, the electric upsetting process can be roughly divided into four stages. (i) The preheating stage, in which the anvil keeps stationary and the portion of billet between anvil and clamping electrode is heated to an elevated temperature. (ii) The primary stage of the forming process, in which the anvil begins to retreat with a given speed, and meanwhile the heating current and upsetting force increase with increasing upsetting cylinder travel distance. (iii) The intermediate stage of the forming process, in which both the heating current and upsetting force reach their maximum, and begin to decrease. (iv) The stable deformation stage, in which both the heating current and upsetting force drop down at a constant rate.

In the present electric upsetting FEM simulation, the preheating time was set as 30 s; and the range of two steps before and after the peak current and force was defined as the intermediate stage of the forming process. The approximate upsetting displacement boundaries for separating the three stages in the forming process are 155 mm and 325 mm according to the parameter loading path diagram. On the other hand, it was found from the numerical simulation results that the electric upsetting time of 350 s and 550 s correspond to the upsetting displacement of 156 mm and 326 mm, respectively. Therefore, for the sake of description, the beginning and end of stage (iii) were specified as 350 s and 550 s. In addition, in this work, the preheating stage, primary stage, intermediate stage, and stable deformation stage are defined as the following four electric upsetting periods:(i) 0~30 s, (ii) 30~350 s, (iii) 350~550 s and (iv) 550~2000 s (the end of the electric upsetting process). A detailed discussion on the temperature field evolution in the four stages will be presented in the following Sections.

## 3. Evolution of Temperature Field in Different Stages of the Electric Upsetting Process

### 3.1. Temperature Field Evolution in the Preheating Stage

**Figure 7:**
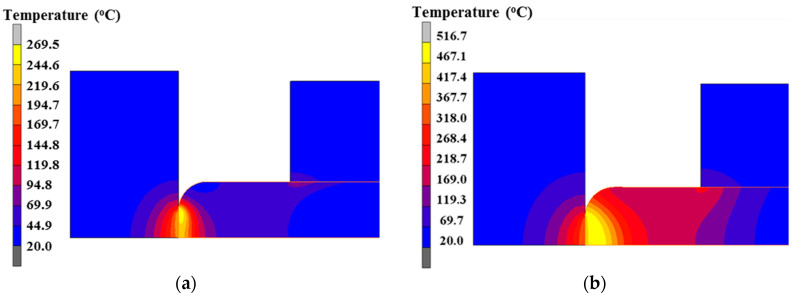
Temperature field at different moments during the preheating process: (**a**) at 5th second and (**b**) at the end moment of the preheating process.

In the first thirty seconds of the electric upsetting process, the anvil remains stationary to ensure the billet is preheated adequately. Figure 7 shows the temperature field distribution of various objects at 5 s and the end moment of the preheating stage. As can be seen from Figure 7, only the portion of billet between anvil and clamping electrode was heated, and the rest of the billet was at room temperature. The bulk temperature of the billet is higher than that of the anvil and clamping electrode. In the initial 5 s, the higher temperature appears at the contact surface between anvil and billet, while the highest temperature shows in the interior of the billet at the end moment of the preheating process. Thermal transfer occurred between the billet and anvil, resulting in the increasing temperature at the contact surface of the anvil. In addition, it can be found that the temperature at the contact surface between billet and clamping electrode is relatively low because more heat is lost to the billet stem rather than to the clamping electrode. This can be explained by the following reasons. Firstly, compared with the thermal transfer between two objects, it is easier to transfer heat within an object because there is no extra contact surface to hinder heat diffusion. On the other hand, for an actual electric upsetting process, the volume of billet stem is much larger than that of clamping electrode. In addition, the specific heat of Ni80A superalloy is larger than that of TZM alloy. Therefore, it is more difficult to heat the billet stem than clamping electrode, thus exerting more heat transferred to the billet stem.

**Figure 8:**
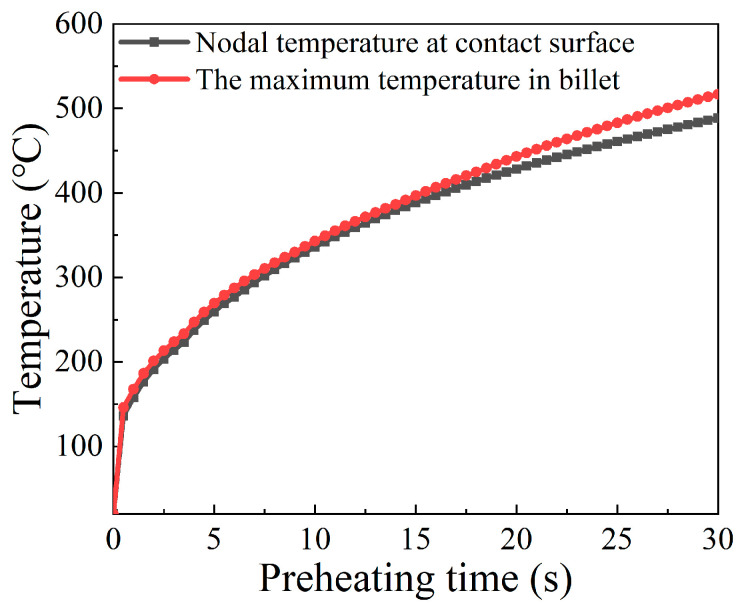
Evolution of the maximum temperature and the nodal temperature of contact surface over time during the preheating stage.

In order to analyze the interior mechanisms that cause such temperature distribution, the highest billet temperature and the nodal temperature on the contact surface varying with preheating time were extracted and plotted in Figure 8. It can be seen that the maximum temperature and contact temperature increase simultaneously with the increasing preheating time. The variation of the temperature at the contact surface is in agreement with that of the maximum temperature at the early stage of the preheating stage, while the rate of increase in contact temperature gradually slows down at the later period of the preheating stage. This implies that the highest temperature moved from the contact surface to the interior of the billet during the preheating process.

**Figure 9:**
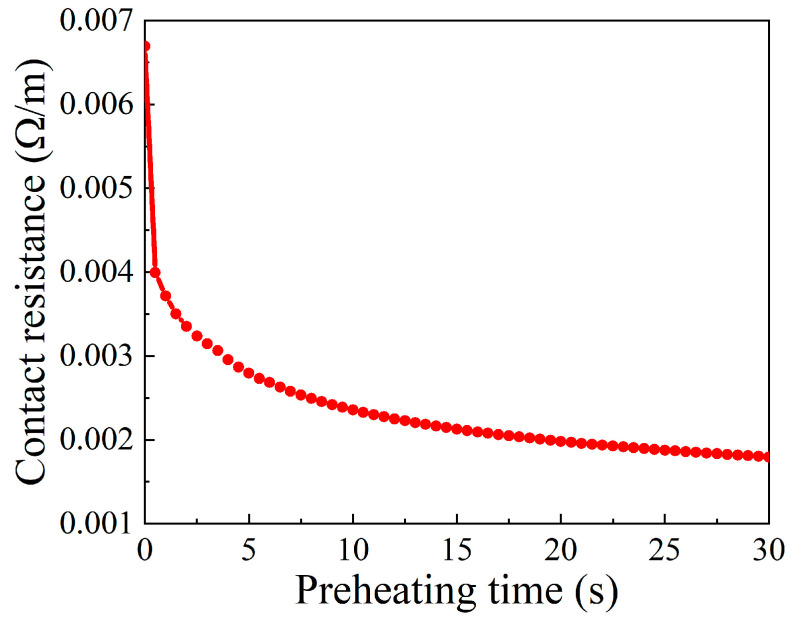
The variation of contact resistance respect to time during the preheating stage.

**Figure 10:**
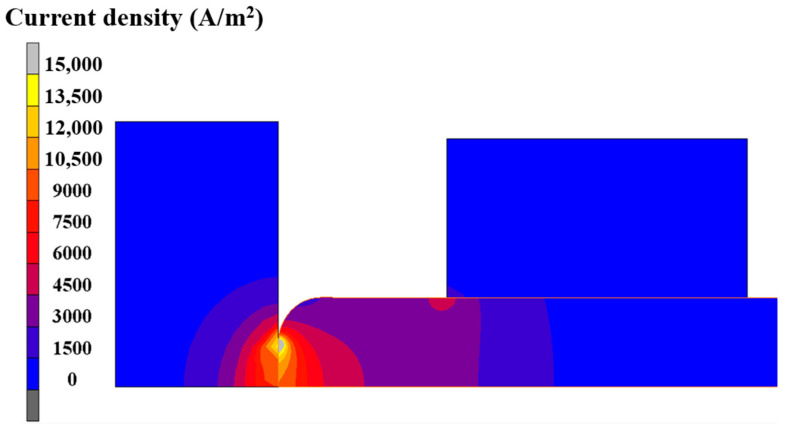
Current density distribution of the electric upsetting system at the end of the preheating process.

Since most heat in the billet comes from electric heating, it is necessary to analyze its current density distribution and electric resistance variation. According to reference [13], the contact resistance can be evaluated by the formula: *R* = 1/(*a*_1_T + *a*_2_), where *T* is the temperature, and *a*_1_ and *a*_2_ are the constants dependent on contact pressure, here 0.87 and 132, respectively. With that, the variation of contact resistance along with preheating time can be exhibited, as in Figure 9. According to Figure 9, the contact resistance decreases with increasing preheating time due to the continuously increasing contact nodal temperature, and the contact resistance function becomes weak at the later stage of the preheating process. In addition, the current density distribution at the end moment of the preheating process was obtained and is revealed in Figure 10. The higher current density is close to the contact surface between billet and anvil because of the relatively lower cross-sectional area at this region. In summary, the reasons accounting for the temperature distribution in the preheating stage are as follows. At the beginning of the preheating stage, the maximum temperature lies at the contact surface because more Joule heat is generated under the comprehensive effects of contact resistance heating and volume resistance heating. Along with the increasing preheating time, the function of contact resistance weakens owing to the elevated contact nodal temperature. By contrast, the thermal conduction between anvil and billet is enhanced with increasing temperature. As a result, abundant heat is lost to the anvil. Owing to the relatively higher current density near the contact surface region of the billet, more Joule heat is generated here. Therefore, under the synthesis effect of Joule heat and thermal conduction, the higher temperature appears in the interior of the billet but close to the contact surface between anvil and billet at the end moment of the preheating stage.

### 3.2. Temperature Field Evolution in the Primary Stage of the Forming Process

**Figure 11:**
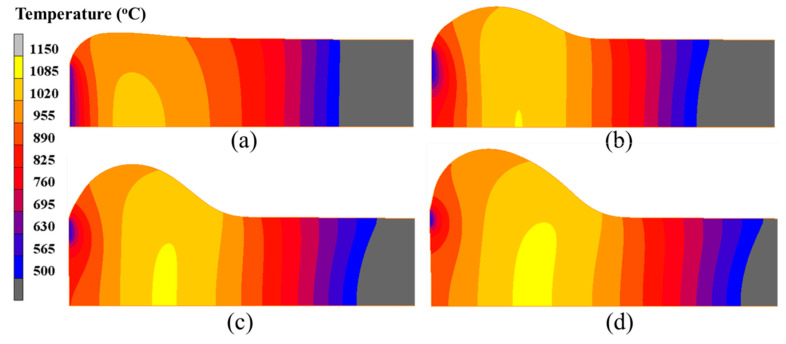
Temperature field distributions in the billet at different moments of the electric upsetting process: (**a**) 200 s, (**b**) 250 s, (**c**) 300 s and (**d**) 350 s.

Figure 11 exhibits the temperature field distribution at different moments during the primary stage of the forming process. It can be noted from Figure 11 that with the increase in heating current and upsetting force, the billet was gradually deformed into an onion shape. Moreover, the onion became longer as the electric upsetting process proceeds. In fact, the plastic deformation of the billet is mainly governed by its temperature field distribution, i.e., the material flow will be more easily accomplished in the higher temperature region. It can be seen from Figure 11 that the higher temperature region has shifted from the interior of the billet to the neck of the onion. Moreover, the main plastic deformation zone also lies in the neck of the onion at the later period of the primary stage, thus forming a long onion shape. In addition, it can be observed from Figure 11 that there exists an obvious low temperature region on the contact surface between anvil and billet, which becomes smaller as the electric upsetting process proceeds. The high temperature region gets larger with the increasing current and force. A clear temperature gradient distribution appears at the right of the onion, which suggests that a lot of heat is lost through the billet stem.

**Figure 12:**
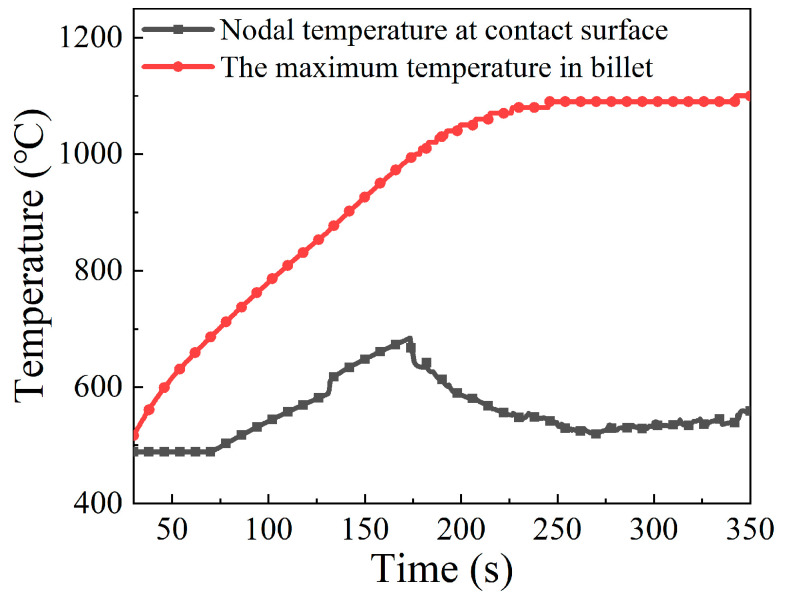
Evolution of the maximum temperature and the nodal temperature of contact surface over time during the primary stage of the forming process.

The evolution of the highest overall temperature and of the contact surface temperature during the primary stage of the forming process is shown in Figure 12. It is apparent that the maximum temperature increased continuously to a plateau because of the increasing current during the primary stage, while the variation tendency of contact temperature is complicated and dynamically changeable. The contact surface temperature increased firstly, and then dropped down at the later period of the primary stage. This may be caused by the fact that with the increasing current in the primary stage, a lot of Joule heat is generated. Meanwhile, the thermal convection coefficient between billet and anvil increased accordingly as the contact surface temperature increased [8]. Finally, the contact surface temperature decreased to an equilibrium state.

**Figure 13:**
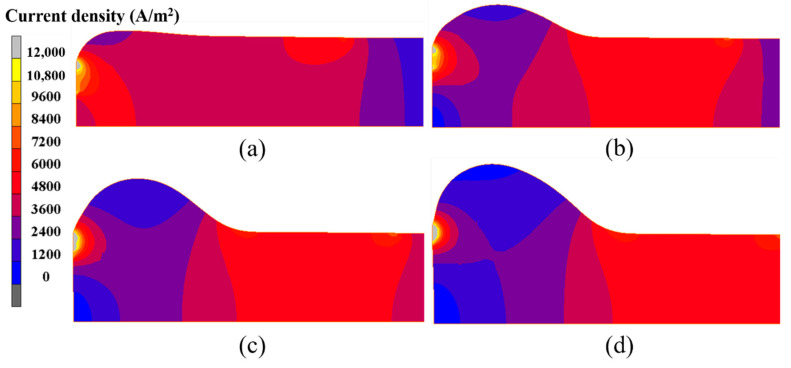
Current density distributions in the billet at different moments of the electric upsetting process: (**a**) 200 s, (**b**) 250 s, (**c**) 300 s and (**d**) 350 s.

Furthermore, the current density evolution in the electric upsetting process was analyzed. The current density distributions at different moments during the primary stage are presented in Figure 13. Clearly, the highest current density appeared at the contact surface. There are two regions, i.e., the bottom center of the billet and the outer surface of the onion, that have relatively lower current density. Generally, in a large-scale electric upsetting process, the formation of an onion is accompanied by the emergence of end-surface depression defect, which makes the contact surface area between anvil and billet smaller. The current always goes through the path with lowest resistance. Therefore, there is a lack of current flow in the two afore-mentioned regions. Correspondingly, little Joule heat was generated here. It is worth noting that the contact surface temperature is still relatively lower although the current density at the contact surface is at its highest level. This is because the material volume for electric heating is relatively small and the effect of thermal conduction is significant. In addition, it can be seen that the neck of the onion also has a relatively high current density, and more Joule heat will be generated here. In a word, the special temperature distribution in the primary stage is induced by the higher current density at the neck of the onion and the stronger thermal conduction between billet and anvil.

### 3.3. Temperature Field Evolution in the Intermediate Stage of the Forming Process

**Figure 14:**
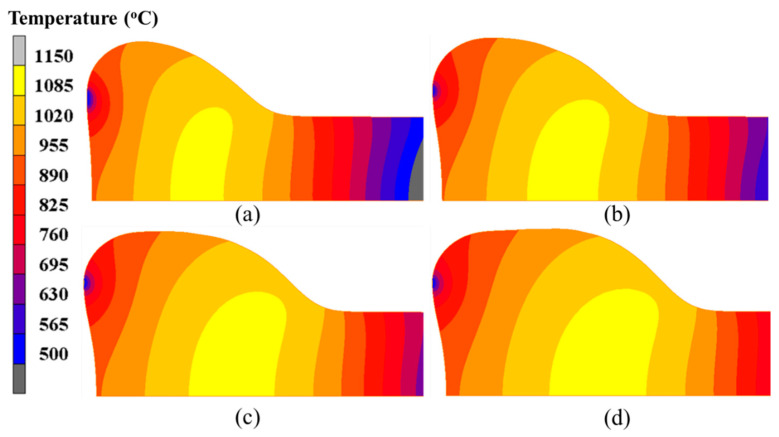
Temperature field distributions in the billet at different moments of the electric upsetting process: (**a**) 400 s, (**b**) 450 s, (**c**) 500 s and (**d**) 550 s.

**Figure 15:**
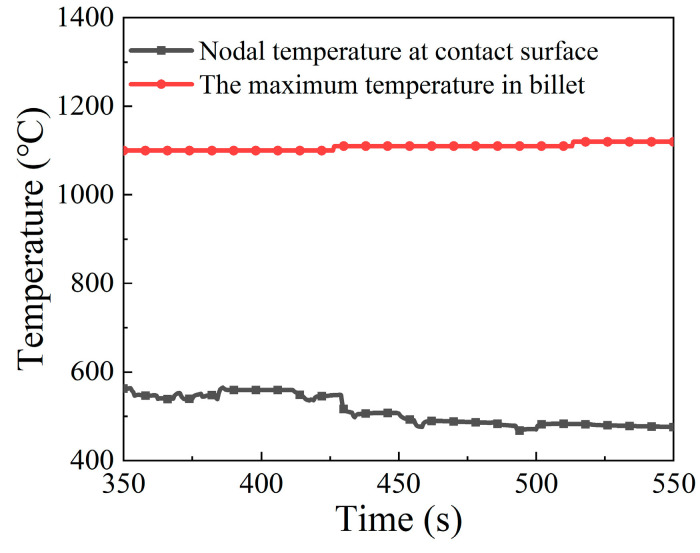
Evolution of the maximum temperature and the nodal temperature of contact surface over time during the intermediate stage of the forming process.

The distributions of the temperature field at different moments in the intermediate stage of the forming process are displayed in Figure 14. A common feature is that the contact surface between billet and anvil shows a relatively low temperature, while the high temperature region always appears in the interior of the billet, and near to the neck of the onion. As the electric upsetting process proceeded, the temperature field changed significantly. The low temperature region began to shrink gradually, while the high temperature region expanded quickly. At the intermediate stage, both the heating current and upsetting force reached their maximums. More heat was generated by resistance heating, thus making the area of high temperature region increase. Meanwhile, the onion was enlarged sufficiently under the high current and upsetting force, which promotes the end-surface depression of the onion. This causes the contact surface between anvil and billet to decrease, thereby resulting in the shrinking of the low temperature region on the billet. In order to quantitatively analyze the evolution of the temperature field during the intermediate stage, the maximum temperature and the contact surface temperature were plotted with respect to electric upsetting time in Figure 15. It can be clearly seen from Figure 15 that the maximum temperature and the contact surface temperature remain basically unchanged during the intermediate stage.

**Figure 16:**
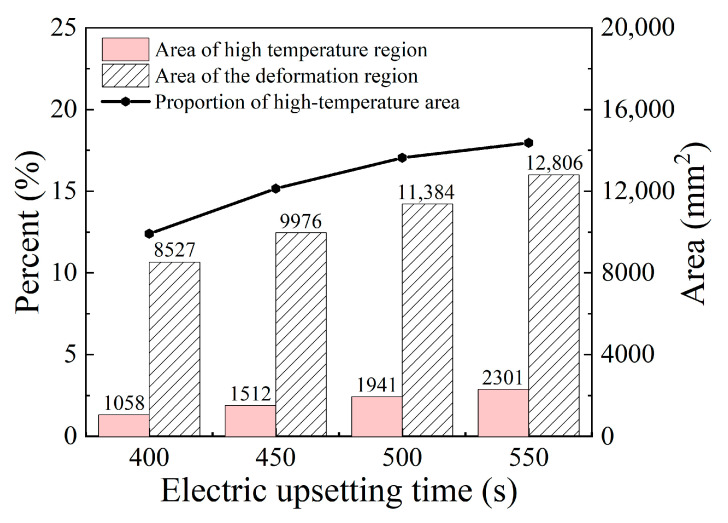
The area of high temperature region and the onion area at different electric upsetting moments during the intermediate stage.

According to our previous investigation, the high temperature region has significant influence on grain growth, and even determines the mechanical properties of the formed workpiece. In order to quantitatively describe the evolution of the high temperature region, the area of high temperature region was evaluated using an indicator *S*. Its calculation method can be expressed as Equation (6),

(6) $$S=\sum_{i=1}^{n}S_{i}$$

where *S_i_* represents the area of every element, and *n* is the number of the elements whose temperature is in the specific range. In this work, the element whose temperature exceeds 500 °C will be identified as representing the onion, while the element whose temperature exceeds 1085 °C will be regarded as representing the high temperature region, and the rest are billet stem elements. The temperature of each element can be estimated using the average value of its four integration point temperatures. Based on the calculation formula, the areas of high temperature region and onion corresponding to the electric upsetting time of 400 s, 450 s, 500 s and 550 s were calculated and revealed in Figure 16. It is observed that the area of high temperature region and its fraction increase simultaneously as the electric upsetting process proceeds during the intermediate stage.

**Figure 17:**
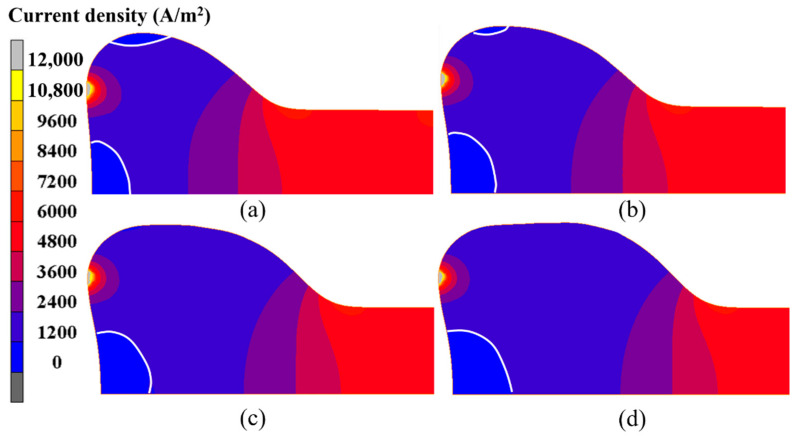
Current density distributions of the billet at different moments of the electric upsetting process: (**a**) 400 s, (**b**) 450 s, (**c**) 500 s and (**d**) 550 s.

According to the above analysis, an interesting phenomenon is that the highest temperature remains basically unchanged, while the high temperature region grows continuously. This result can be explained by the following reasons. During the intermediate stage of the electric upsetting, the astern speed of the anvil is constant, and the variations of heating current and upsetting force are small, so that the electric upsetting system is always in equilibrium. Correspondingly, the highest temperature remains relatively constant. As for the size of the high temperature region, this is closely associated with the current density distribution of the billet. Here, the current density distributions of the billet corresponding to the electric upsetting time of 400 s, 450 s, 500 s and 550 s are presented in Figure 17, in which the regions without current flow are indicated by white curves. It is apparent that the distribution of current density becomes more and more homogeneous as the electric upsetting process proceeds. Moreover, the region circled by white curves near the outer surface of the onion begins gradually to shrink and finally disappears. Consequently, the main reason for the enlarged high temperature region can be attributed to the increasingly homogeneous current density distribution.

### 3.4. Temperature Field Evolution in the Stable Deformation Stage

**Figure 18:**
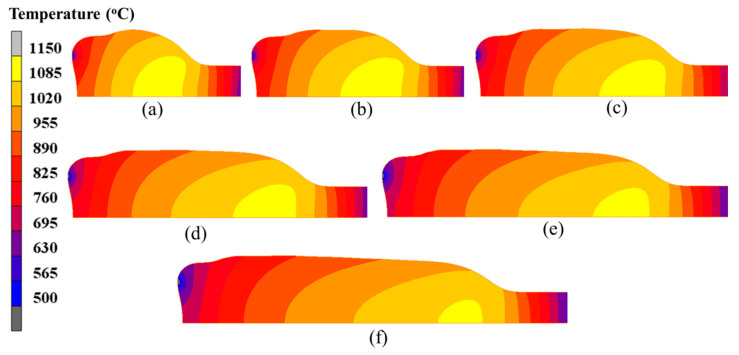
Temperature field distributions of the billet at different electric upsetting moments: (**a**) 750 s, (**b**) 1000 s, (**c**) 1250 s, (**d**) 1500 s, (**e**) 1750 s and (**f**) 2000 s.

Figure 18 shows the temperature distribution of the billet at 750 s, 1000 s, 1250 s, 1500 s, 1750 s and 2000 s. During the stable deformation stage, the onion became very long, and the onion diameter was gradually decreased as the electric upsetting process proceeded. All the temperature distributions in Figure 13 show similar characteristics, i.e., the contact surface between anvil and billet has relatively lower temperature, and the high temperature region always exists in the interior of the billet but close to the onion’s neck. In addition, an obvious temperature gradient distribution is observed: from the interior to the outer surface of billet, the temperature decreases gradually; from the left end-surface to the stem part of the billet, the temperature firstly increases and then decreases.

**Figure 19:**
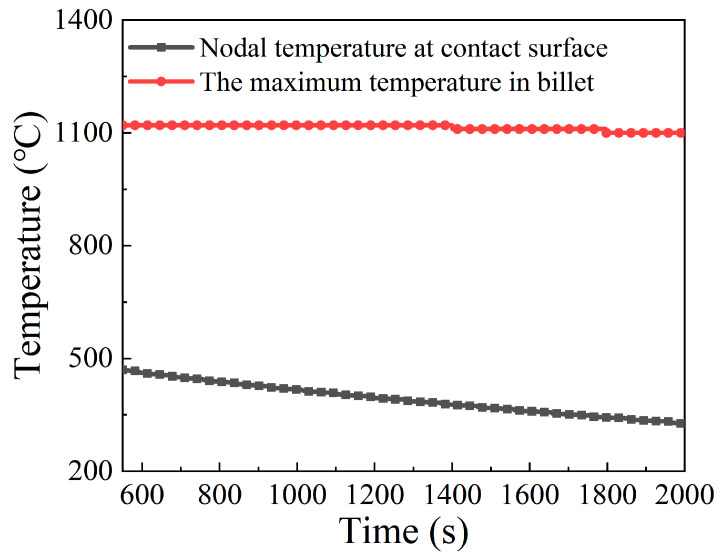
Evolution of the maximum temperature and the nodal temperature of contact surface along with electric upsetting time during the stable deformation stage.

**Figure 20:**
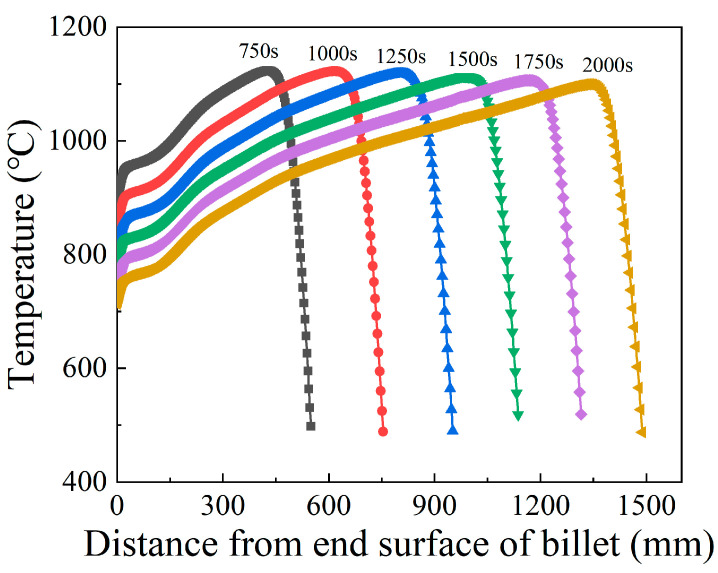
Temperature distributions on the central axis of the billet corresponding to diverse electric upsetting moments.

In order to quantitatively analyze the temperature distribution during the stable deformation stage, the evolution of maximum temperature and of contact surface temperature with respect to electric upsetting time were extracted and are presented in Figure 19. Apparently, the maximum temperature remains relatively constant during the stable deformation stage, and the contact surface temperature keeps decreasing slowly. Although the decreasing heating current during the stable deformation stage may lead to less Joule heat generated by electric heating, it can simultaneously decrease the diameter of the onion [10], thus by contrast leading to a relatively higher current density at the neck of the onion. Thus, under the self-coordination of current density and plastic deformation, the maximum temperature remained unchanged. As for the continuously decreasing contact surface temperature, this may be attributed to the gradually decreasing heating current. During the stable deformation stage, the heating current decreases, and the contact surface area between billet and anvil remains unchanged according to Figure 18. In this case, the current passing through the contact surface would reduce, and the Joule heat generated at the contact surface decrease accordingly. Moreover, the temperature distributions on the central axis of the billet corresponding to diverse electric upsetting moments were obtained and are revealed in Figure 20. The *X*-axis in Figure 20 represents the distance away from the left end-surface of the onion. It can be apparently observed that the variation tendency of these curves is very similar. In addition, it can be found from Figure 20 that the length of onion and the position of the highest temperature are linearly correlated with the electric upsetting time, which also indicates that the forming process of the onion is in a relatively steady state during the stable deformation stage.

**Figure 21:**
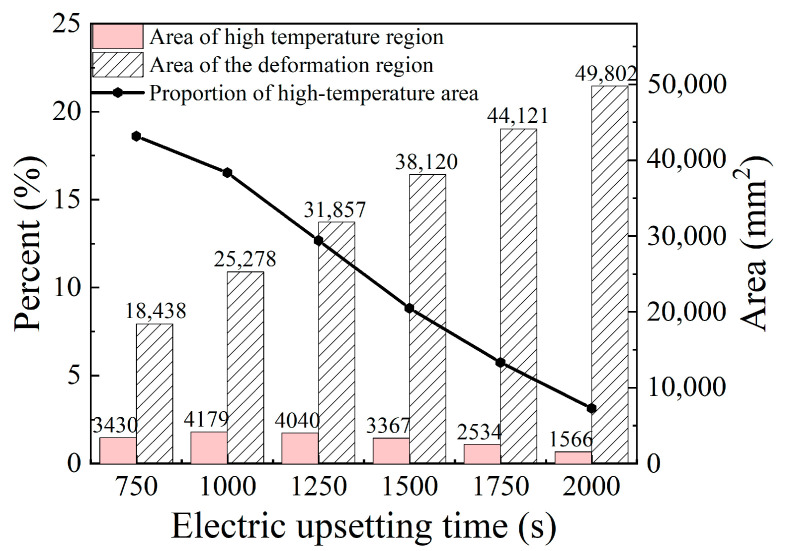
The area of the high temperature region and the onion area corresponding to diverse electric upsetting moments during the stable deformation stage.

For the sake of analyzing the evolution of the high temperature region, the area of high temperature region and the area of the deformation region (area of the onion) at the electric upsetting moments of 750 s, 1000 s, 1250 s, 1500 s, 1750 s and 2000 s were counted and shown in Figure 21. It can be seen that the proportion of the high temperature region decreases continuously as the electric upsetting process proceeds. Particularly, the area of the high-temperature region increases during the electric upsetting period of 750~1000 s while its area fraction decreases. This may be caused by the following reasons. During the electric upsetting period of 750~1000 s, both the heating current and the upsetting force are still in a relatively high level. The area of the high temperature region increases under the effect of the high current. On the other hand, the area of the onion also increases owing to the relatively high current and strong upsetting force. Moreover, the increasing rate of the onion’s area is much higher than that of the high temperature region. That is why the area of high temperature increases and its area fraction decreases during the period 750~1000 s.

**Figure 22:**
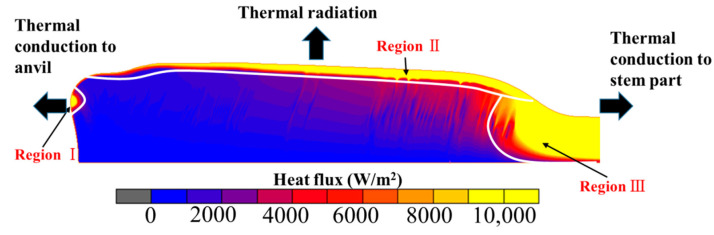
Heat flux distribution of billet at the end moment of the electric upsetting process.

In order to clarify the formation mechanisms of these temperature distributions in the stable deformation stage, the heat flux distribution of the billet is presented in Figure 22. It can be clearly seen that there are three regions with relatively higher heat flux. For region I, thermal conduction between anvil and billet is significant, as analyzed in Section 3.1 and Section 3.2. It is well known that thermal radiation generally acts on the surface of objects. Therefore, for region Ⅱ, the main heat loss mechanism is thermal radiation. As for region Ⅲ, it is close to the neck of the onion where abundant Joule heat is generated. Moreover, most heat was transferred to the newly-fed material, i.e., the part of the billet stem in this region. In a word, due to the intense thermal conduction between anvil and billet, strong thermal dispersion to the billet stem, abundant Joule heat generated at the neck of the onion, as well as the remarkable thermal radiation on the outer surface of the onion, the unique temperature distributions in the stable deformation stage of the electric upsetting process is formed.

## 4. Verification Based on Electric Upsetting Experiment

In order to verify the established electric-thermal-mechanical multi-field coupling electric upsetting FEM model, a practical electric upsetting experiment was conducted on an electric upsetting machine using the loading parameters exhibited in Figure 6. To ensure superior wear-resistance, TZM alloy is usually employed as the anvil and clamping electrode material. The trail process was recorded by a digital camera. A fixed infrared temperature measuring device was mounted on the electric upsetting machine to monitor and record the temperature variation of the billet. For the preheating process, the point temperature at the fillet of the billet was measured, and the temperature data were recorded every 5 s. For the forming process, the point temperature at the onion neck was monitored, and the temperature data were recorded every 50 s.

**Figure 23:**
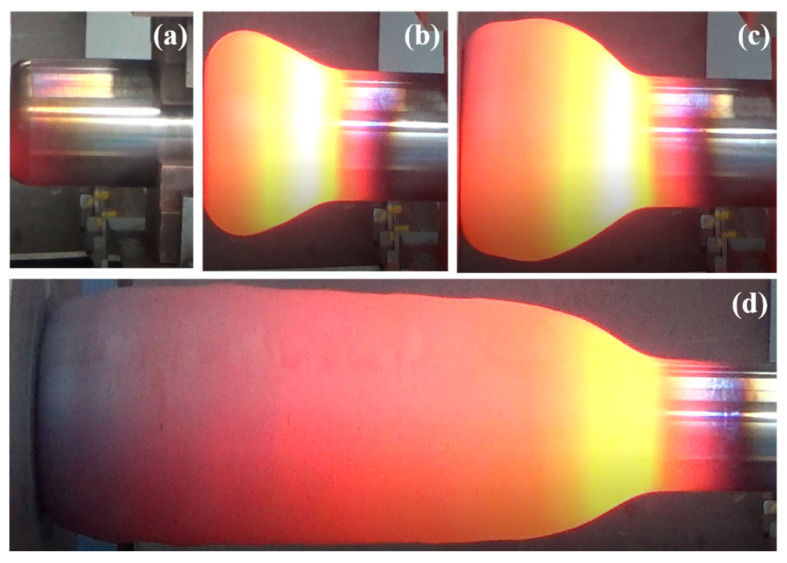
Trial process of large-scale electric upsetting at different stages: (**a**) the preheating stage; (**b**) the primary stage of forming process; (**c**) the intermediate stage of forming process; (**d**) the completed state.

Figure 23 shows the forming processes of the billet at the end moments of the four stages. It can be seen from Figure 23 that the shape of the onion and position of the higher temperature region are consistent with the numerical simulation results. At the preheating stage, the higher temperature appears in the left end-part of the billet, which is in agreement with the simulation results (Figure 7). For the following three stages of the forming process, the neck of the onion is clearly brighter than other parts (Figure 23b–d), which implies a higher surface temperature at the neck of the onion. This is also consistent with the simulated temperature distribution. In addition, the length and the maximum diameter of the onion formed in the electric upsetting experiment were measured as 362 mm and 148 mm, respectively. The corresponding values obtained from numerical simulation are 367 mm and 144 mm, respectively. Thus, the longitudinal and radial errors between the simulated onion shape and the experimental one are estimated as 1.38% and 2.70%, respectively.

**Figure 24:**
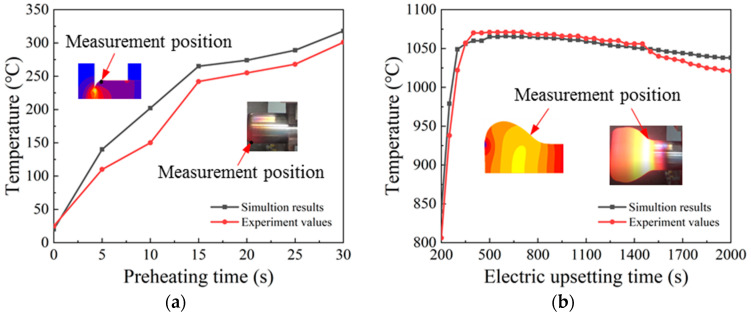
Comparison between simulation temperature and experimental one during different processes: (**a**) the preheating process, and (**b**) the forming process.

Both of the temperature data obtained from the electric upsetting experiment and from the finite element simulation were plotted in Figure 24. Figure 24a shows the temperature comparison results at the preheating stage. It is noted that the temperature difference between simulation and experiment firstly increases, and then decreases at the later period of the preheating stage. This may be caused by the sudden variation of upsetting force. At the beginning of the electric upsetting process, the upsetting force increases from zero to a specific value, thus making the disturbance of the electric upsetting system intensify. Figure 24b shows the comparison of temperature in the forming process. It is obvious that the temperature difference is larger at the early and last period of the forming process. Moreover, the variations of upsetting force and heating current are small at the stable deformation stage, thereby resulting in a relatively constant temperature variation. The temperature difference is accordingly small. At the later period of the electric upsetting process, the onion volume has become huge, and the effect of gravity on the deformation process and the temperature distribution of the onion increases. Therefore, the difference between simulated temperature and experimental temperature is gradually widened at the later period of the electric upsetting process.

In order to justify the accuracy of the established electric upsetting FEM model, the average relative error between simulated temperature data and experimental ones will be evaluated by Equation (7),

(7) $$ARE=\frac{1}{n}\sum_{i=1}^{n}\left|\frac{T_{i}^{S}-T_{i}^{E}}{T_{i}^{E}}\right|$$

where *ARE* represents the average relative error; $T_{i}^{S}$ represents the simulated temperature data; $T_{i}^{E}$ represents the experimental temperature data; and *n* is the number of recorded temperature data. For the preheating stage, *n* is 7; for the forming process, *n* is 36. Therefore, the finial *ARE*-value of the electric upsetting process is calculated as 7.54%. According to the comparison of simulated temperature data and experimental ones in Figure 24, it can be concluded that the simulated temperature variation is in good agreement with the experimental one, which indicates that the developed electric upsetting FEM model is credible.

## 5. Conclusions

In this work, an electric-thermal-mechanical multi-field coupling FEM model was established to simulate the electric upsetting process. The distribution characteristics of the temperature field in different stages of the electric upsetting process, and the field formation mechanisms, were analyzed in detail. This work provides a thorough insight into the temperature evolution in the electric upsetting process. It contributes to future work on predicting the microstructure evolution and analyzing the defect formation during the electric upsetting process. Moreover, it offers an important guidance for future work on finding an appropriate method to adjust the temperature distribution in electric upsetting. Several conclusions can be drawn from this work as follows.

(1) During the preheating process, the higher temperature firstly appears at the contact surface between anvil and billet due to the combined effects of contact resistance and volume resistance. With increasing preheating time, the higher temperature is transferred to the interior of the billet because the effect of contact resistance weakens with increasing temperature.
(2) For the primary stage of the forming process, the high temperature region is transferred from the interior of the billet to the neck of the onion, and an obvious low temperature region emerges at the contact surface between anvil and billet because the effect of heat transfer between anvil and billet is enhanced under the elevated temperature, and the relatively higher current density concentrates in the neck of the onion.
(3) In the intermediate stage of the forming process, the high temperature region always appears at the neck of the onion, and the lower temperature region is at the contact surface. In addition, the area and proportion of high temperature region both increase as the electric upsetting process proceeds, which is attributed to the increasingly homogenous current density distribution.
(4) As for the stable deformation stage of the electric upsetting process, the high temperature region still appears at the neck of the onion, and the low temperature regions lie in the contact surface and the outer surface of the onion owing to the heat transfer between anvil and billet, thermal radiation on the outer surface of the billet, and thermal dispersion to the billet stem, as well as abundant Joule heat generated at the neck of the onion.

## Figures and Tables

**Table 1:** Chemical composition of Ni80A superalloy.

Element	Al	Si	Mn	Cr	Ti	Fe	C	Ni
Composition (wt%)	0.68	0.55	0.63	20.87	2.07	1.26	0.069	Bal.

**Table 2:** Chemical composition of TZM alloy.

Element	Ti	Zr	C	Fe	Ni	Si	N	Mo
Composition (wt%)	0.5	0.08	0.02	0.005	0.002	0.002	0.001	Bal.

**Table 3:** Physical properties of Ni80A superalloy and TZM alloy.

		Temperature (°C)					
		300	500	700	900	1100	1200
Ni80A	*C* (J/g·°C)	0.519	0.573	0.628	0.601	0.678	0.755
	*λ* (W/m·°C)	16.1	19.4	22.3	26.5	29.8	31.5
	*ρ* (g/m^3^)	8190					
TZM	*C* (J/g·°C)	0.250	0.265	0.274	0.291	0.300	—
	*λ* (W/m·°C)	24.6	25.0	25.5	26.0	26.0	26.0
	*ρ* (g/m^3^)	10200					

where *C* is specific heat, *λ* is heat conductivity, and *ρ* is mass density.

## Data Availability

Not applicable.
